# Are Changes in Physical Work Capacity Induced by High-Intensity Functional Training Related to Changes in Associated Physiologic Measures?

**DOI:** 10.3390/sports6020026

**Published:** 2018-03-27

**Authors:** Derek A. Crawford, Nicholas B. Drake, Michael J. Carper, Justin DeBlauw, Katie M. Heinrich

**Affiliations:** 1 Department of Health, Human Performance, and Recreation, Pittsburg State University, Pittsburg, KS 66763, USA; nbdrake@gus.pittstate.edu (N.B.D.); mjcarper@pittstate.edu (M.J.C.); 2 Functional Intensity Training Laboratory, Department of Kinesiology, Kansas State University, Manhattan, KS 66506, USA; jdeblauw@ksu.edu (J.D.); kmhphd@ksu.edu (K.M.H.)

**Keywords:** High-Intensity Functional Training, work capacity, performance

## Abstract

High-Intensity Functional Training (HIFT) is a novel exercise intervention that may test body systems in a balanced and integrated fashion by challenging individuals’ abilities to complete mechanical work. However, research has not previously determined if physical work capacity is unique to traditional physiologic measures of fitness. Twenty-five healthy men and women completed a six-week HIFT intervention with physical work capacity and various physiologic measures of fitness assessed pre- and post-intervention. At baseline, these physiologic measures of fitness (e.g., aerobic capacity) were significantly associated with physical work capacity and this relationship was even stronger at post-intervention assessment. Further, there were significant improvements across these physiologic measures in response to the delivered intervention. However, the change in these physiologic measures failed to predict the change in physical work capacity induced via HIFT. These findings point to the potential utility of HIFT as a unique challenge to individuals’ physiology beyond traditional resistance or aerobic training. Elucidating the translational impact of increasing work capacity via HIFT may be of great interest to health and fitness practitioners ranging from strength/conditioning coaches to physical therapists.

## 1. Introduction

High-Intensity Functional Training (HIFT) is currently one of the fastest growing fitness trends in the world [1]. Part of the reason for this popularity is HIFT’s demonstrated efficacy for a wide range of health and fitness measures ranging from improvements in body composition to aerobic capacity [2,3,4,5,6]. However, recent work shows that the magnitude of these effects on body structures and functions may be rather modest in nature with potentially differing directions [7]. Despite modest and potentially inconsistent effects on fitness components, HIFT appears to have a large impact on an individual’s ability to perform physical work [7]. 

Physical work capacity represents an individual’s ability to complete a maximal amount (e.g., volume) of mechanical work across differing modalities, intensities, and time domains using the appropriate bioenergetic pathways (i.e., phosphocreatine, glycolytic, and oxidative) [8]. HIFT challenges physical work capacity through four mechanisms: (1) by addressing multiple fitness domains (e.g., aerobic and resistance training) [9], (2) in emphasizing functional exercises that require universal motor patterns (e.g., pushing and squatting) [10], (3) by temporally combining aerobic and resistance training elements within exercise sessions [11,12], and (4) with consistent focus on high effort or intensity [8]. Further, these mechanisms are incorporated into training sessions in variable patterns across multiple time domains (i.e., short and long durations), creating a unique stimulus virtually every day. 

Butcher et al. [13] postulate that challenging physical work capacity may represent a unique exercise stimulus beyond traditional exercise programs. One reason that HIFT may represent a novel challenge to body homeostasis (i.e., the constant and normal internal environment during resting conditions) is the unique structure and implementation of the training program. Rather than training a single component of fitness (e.g., muscular strength) in relative isolation, HIFT requires multiple body systems to work together in a maximal, balanced, and integrated fashion throughout training sessions. However, to-date, no investigations have tested this hypothesis. 

In contrast, one could reasonably assume that possessing a high level of proficiency in all physiological components of fitness (e.g., aerobic and anaerobic capacity, etc.) would enable a high level of work capacity performance. In fact, Butcher et al. [13] demonstrated that aerobic capacity and lower extremity muscular strength successfully predicted acute HIFT performance. However, demonstration of an association between baseline physiology and performance is not equivalent to establishing a cause-and-effect relationship resulting from a training intervention [14]. Thus, we cannot assume that changes in components of fitness induced by HIFT are the cause of individual work capacity change. 

With this in mind, the purpose of the present study was to determine the relationship between the change in various physiologic measures of fitness and the change in physical work capacity resulting from a HIFT intervention. We hypothesized that the HIFT intervention would cause significant improvement across various physiologic measures of fitness (e.g., lower extremity muscular strength) and that pre-intervention values for these measures would be moderately correlated to work capacity at baseline. However, despite these improvements and baseline association, we also hypothesized that any improvement in physical work capacity from the HIFT intervention would be independent of changes in the associated physiologic measures of fitness. 

## 2. Materials and Methods

### 2.1. Participants

Twenty-five healthy men (*n* = 13; Mn age = 22.6 ± 3.5; Mn body mass = 86.1 ± 13.9 kg; Mn height = 182.8 ± 8.1 cm) and women (*n* = 12; Mn age = 21.0 ± 1.5; Mn body mass = 70.5 ± 11.3 kg; Mn height = 165.6 ± 5.7 cm) agreed to participate in the study. Participants were required to be untrained as defined by not pursuing any specific health or fitness goal (e.g., weight loss or improving aerobic capacity) at least six months prior to study commencement yet could be recreationally active. All participants reported no significant disease or health conditions (e.g., peripheral artery disease) that might have been a contraindication for vigorous exercise. Written informed consent was obtained from all participants prior to study commencement and all procedures were approved by a University Institutional Review Board for the Protection of Human Research Subjects. 

### 2.2. Experimental Design

This study was carried out over a nine-week period to determine the association of HIFT-induced changes in physiologic measures of fitness and changes in physical work capacity. Outcomes were measured at baseline and nine weeks (i.e., post-test) for two days each week with 48 h between each testing session. Training was performed during weeks two, three, four, six, seven, and eight for five days on (Monday–Friday) and two days off (Saturday and Sunday) each week. Thus, participants were asked to attend 36 (six testing and 30 training) sessions. Several training times were offered each day to accommodate all participants while maintaining a safe participant-to-instructor ratio. All sessions were supervised and guided by a trained masters-level university student with a CrossFit Level 1 certificate. 

### 2.3. High-Intensity Functional Training Intervention 

The HIFT intervention protocol used within the present study followed the CrossFit (CrossFit, Inc., Washington, DC, USA) template (see Appendix A, Table A1) [8]. All training sessions were held at a local facility that was conducive to the training needs (i.e., a facility with equipment and space for the workouts). Exact details for each training session’s Workout of the Day (WOD) structure and included elements can be found in Table A1. Each training session lasted approximately 60 min including a warm-up period, WOD, and a cool-down. Prior research has shown a minimum dose of 16 HIFT sessions over a three-to-five week period is needed to provide significant effects on various body structures and functions [7,11,12]. Thus, for the present study double the minimum effective dose (i.e., 30 sessions) was selected in an attempt to ensure significant changes in outcome measures were observed. Participants were asked to refrain from all exercise activity outside of the study but remained in free-living conditions. 

### 2.4. Aerobic Capacity 

Aerobic capacity (VO_2max_) for each participant was assessed via the Bruce Treadmill Test [15]. A regression equation based on time to completion for the test was used to determine VO_2max_ [16]. The standard error of the estimate for males was ±3.35 mL/kg^−1^/min^−1^ and ±2.70 mL/kg^−1^/min^−1^ for females.

### 2.5. Anaerobic Capacity 

Anaerobic capacity was assessed via the Wingate Anaerobic Test [17] on a cycle ergometer (Monark 894 E, Monark, Sweden). Primary outcomes of interest were peak power (Power) and fatigue index (FI; % decline in power). Raw data collected by the cycle ergometer was immediately analyzed by software provided by the ergometer manufacturer (Monark Anaerobic Test Software v. 2.0, Monark, Sweden). 

### 2.6. Maximal Strength 

Maximal strength was determined using a standard one-repetition maximum (1RM) protocol for both lower and upper extremity exercises [18]. The exercises utilized were the back squat (Sq), strict shoulder press (P), and conventional deadlift (DL). Each lift was supervised by the trained graduate student, and participants’ rest times were allowed to be no less than three minutes and no more than five minutes between sets. 

### 2.7. Work Capacity 

Physical work capacity was assessed by recording participants’ performance on a selected WOD during week two (i.e., Day 3 in Table A1) and week eight (i.e., Day 28 in Table A1). Assessment occurred within the intervention to “blind” participants to this outcome measure. By “blinding” participants to when this variable was being assessed the authors hoped to minimize any potential confounding factors of performance (e.g., motivation or outcome expectancies). This WOD was designed by study investigators D.C. and N.B.D. so that it would minimize bias toward participants with high levels of gymnastics skill. Further, the time duration (i.e., 10 min) selected for this WOD was such to balance between short (i.e., predominately anaerobic) and long duration (i.e., predominately aerobic) efforts. Participants’ performance was monitored at each attempt by a trained research assistant and participants’ performance was scored as the total number of repetitions (i.e., volume) of all elements/movements completed during the 10-minute period (e.g., 48 repetitions per round × 3 rounds completed = 144 repetitions). 

### 2.8. Statistical Analyses 

Prior to performing inferential analyses, all data were tested for normality and descriptive statistics were calculated. Pearson r correlation coefficients were derived between all study outcome variables at both pre- and post-intervention. A repeated measures MANOVA including all study outcome variables was used to detect mean differences between pre- and post-intervention time points. Significant multivariate effects were followed up with separate paired-samples *t*-tests. Multiple linear regression was used to determine the relationship between the change in significantly correlated fitness components (i.e., VO_2max_, Sq, Power, DL, and PP) and the change in physical work capacity following the HIFT intervention. All analyses were conducted using SPSS version 24.0 for Windows (IBM, Armonk, NY, USA). An alpha level of 0.05 was used for all null hypothesis testing. Supporting statistical information including *p*-value (*p*), effect size (ES), 95% confidence interval (95% CI), and observed power (OP) were reported where appropriate.

## 3. Results

### 3.1. Intervention Adherence 

The mean adherence rate for participants in the HIFT intervention was 87.9 ± 8.3% of the 30 training sessions. There was no significant difference in adherence rate between male (Mn = 87.9 ± 7.8%) and female (Mn = 88.0 ± 9.2%) participants (*t* = −0.031; *p* = 0.976; Mn difference = −0.10%; 95% CI = −7.20, 6.99). 

### 3.2. Baseline Relationships 

Table 1 shows the correlation coefficients for all primary study outcome variables. At baseline, there were significant associations between four out of five predictor variables (VO_2max_, Sq, P, DL, and Power) and work capacity. Only FI was not significantly associated with work capacity at baseline. Post-intervention, all baseline associations between predictor variables and work capacity remained significant while also the strength of the relationships increased. Additionally, there were significant associations post-intervention that were not present at baseline. Namely, the associations between aerobic capacity and maximal strength outcomes (i.e., Sq, P, and DL).

### 3.3. Effects on Physiologic Measures of Fitness

**Figure 1:**
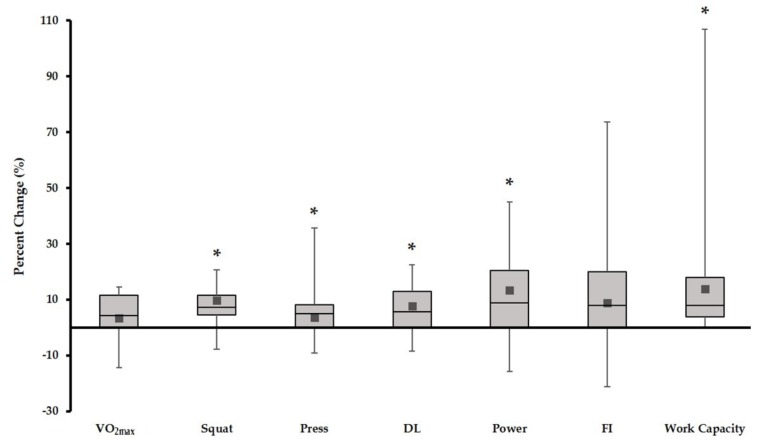
Percent change scores across all primary study outcome variables. * pre-post mean values significantly different at *p* < 0.05.

Figure 1 illustrates the percent change across all primary study outcome variables. This representation was chosen to give readers the complete picture with respect to individual-level change in the variables assessed. In this figure, the box represents the 25th percentile, median, and 75th percentile of change. The error bars represent the minimum and maximum effects observed with the black square denoting the mean change. 

#### 3.3.1. Aerobic Capacity 

Baseline aerobic capacity (Mn = 44.2 ± 2.7 mL/kg^−1^/min^−1^) was not significantly different from post-intervention measurement (Mn = 45.8 ± 3.0 mL/kg^−1^/min^−1^) (*F* = 3.51; *p* = 0.07; Mn difference = 1.60; 95% CI = −0.19, 3.39; ES = 0.163; OP = 0.427). However, the mean percent change from baseline to post-intervention of +3.3% remained outside measurement error typically associated with direct assessment of pulmonary gas exchange [19]. 

#### 3.3.2. Anaerobic Capacity 

There was a significant difference in peak anaerobic power pre- (Mn = 670.2 ± 112.1 W) to post-intervention (Mn = 723.0 ± 117.6 W) (*F* = 6.36; *p* = 0.021; Mn difference = 57.2 W; 95% CI = 8.83, 96.73; ES = 0.261; OP = 0.665). The mean percent change in peak power was +13.4%. In contrast, there was no significant difference in the fatigue index of anaerobic capacity pre- (Mn = 57.4 ± 4.5%) to post-intervention (Mn = 59.6 ± 3.1%) (*F* = 1.01; *p* = 0.327; Mn difference = 2.19%; 95% CI = −2.38, 6.77; ES = 0.053; OP = 0.159). The mean percent change in fatigue index was +8.8%. 

#### 3.3.3. Maximal Strength 

Pre- (Mn = 102.9 ± 9.2 kg) to post-intervention (Mn = 110.8 ± 9.5 kg) there was a significant increase in squat 1RM (*F* = 27.7; *p* < 0.001; Mn difference = 7.92 kg; 95% CI = 4.76, 11.09; ES = 0.606; OP = 0.999). The mean percent change in maximal squat performance was +9.8%. There was a significant difference in press 1RM pre- (Mn = 47.5 ± 4.9 kg) to post-intervention (Mn = 49.6 ± 5.1 kg) (*F* = 5.76; *p* = 0.027; Mn difference = 2.0 kg; 95% CI = 0.26, 3.91; ES = 0.242; OP = 0.662). The mean change in maximal press performance was +3.6%. There was a significant pre- (Mn = 122.6 ± 20.5 kg) to post-intervention (Mn = 130.5 ± 22.5 kg) difference in deadlift 1RM (*F* = 12.27; p = 0.003; Mn difference = 7.9 kg; 95% CI = 3.17; 12.68; ES = 0.405; OP = 0.912). The mean percent change for maximal deadlift performance was +7.6%. 

#### 3.3.4. Work Capacity 

There was a significant increase in physical work capacity from pre- (Mn = 138.3 ± 13.1 reps) to post-intervention (Mn = 153.5 ± 12.4 reps) (*F* = 16.12; *p* = 0.001; Mn difference = 15.2 reps; 95% CI = 7.33 22.91; ES = 0.412; OP = 0.970). The mean percent change in work capacity performance was +13.8%. 

### 3.4. Relationship of Change in Physiologic Measures of Fitness and Change in Work Capacity 

Table 2 displays statistical data for the parameters of a multiple regression model using the associated components of fitness to predict the change in physical work capacity controlling for gender. As shown, the overall model does not significantly predict the change in work capacity induced by HIFT (*F* = 0.330; Sum of Squares = 637.3; *df* = 5; Mean Square = 106.2; *p* = 0.908). Further, within the overall model, no single entered variable significantly predicted the change in work capacity. 

**Figure 2:**
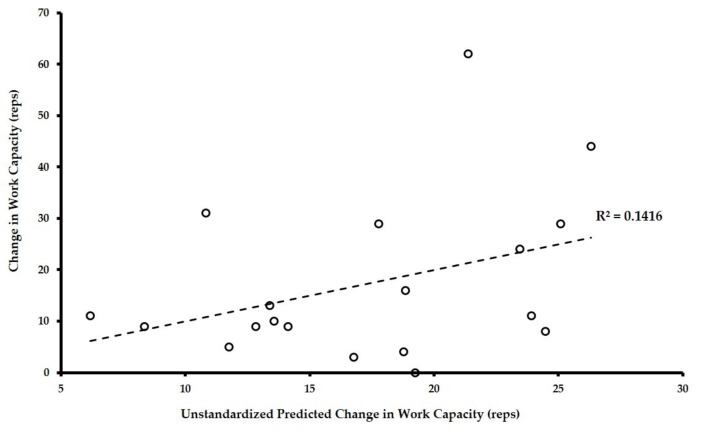
Actual versus predicted change in work capacity from derived multiple regression equation.

Figure 2 shows the scatterplot data for the actual change in work capacity versus the predicted change in work capacity for the multiple regression model outlined in Table 2. 

As illustrated in Figure 2, the regression model tested only accounted for approximately 14% of the variance in the change in individuals’ work capacity. With 86% of the variation unaccounted for, change in work capacity was largely independent of the change in its associated components of fitness. The effect size (ES = 0.165) and statistical power (OP = 0.203) for the overall regression model were calculated using the statistical program R version 3.4.1 (R Statistical Computing Software v. 3.4.4, The R Foundation, Vienna, Austria). Further diagnostic analyses revealed the model did not have issues with multicollinearity (i.e., calculated variance inflation factor statistics all ranged between 1–5) or heteroscedasticity (non-significant Glejser test of unstandardized residuals for all predictor variables). Testing the potential of alternative models, backward model selection (i.e., removing the least significant predictor variable and re-running the regression analysis) revealed the presence of no more parsimonious models to predict the change in work capacity. 

## 4. Discussion

As stated, we hypothesized that the HIFT intervention would cause significant improvement across various physiologic measures of fitness and that pre-intervention values for these measures would be correlated to work capacity at baseline. However, despite these improvements and baseline association, we also hypothesized that any improvement in physical work capacity from the HIFT intervention would be independent of changes in the associated physiologic measures of fitness. The results of this work show support our hypotheses in that physiologic measures of fitness are associated with work capacity performance at baseline and post-intervention. Further, the HIFT intervention employed significantly improved several of the physiologic measures assessed during this study. However, despite these associations and significant improvements, the physiologic fitness measures largely failed to predict the change in individuals’ physical work capacity in response to the HIFT intervention. 

Prior work on HIFT shows that there are significant associations between physiologic measures of fitness and work capacity performance [13,20]. Butcher et al. [13] show that whole-body muscular strength successfully predicts CrossFit-related work capacity performance, which others have also demonstrated [20]. In contrast to these studies, our findings show that there are significant associations between aerobic (i.e., VO_2max_) and anaerobic capacity (i.e., peak power), in addition to whole-body muscular strength, with work capacity performance. Findings from Bellar et al. [21] support these associations as they also show aerobic and anaerobic capacity relate to select modes (i.e., WOD selection and/or style) of work capacity performance. One reason for these differences in association could be the homogeneity of the respective study populations. While the previous studies collected data from competitive CrossFit athletes, the present study included only recreationally active participants. It is plausible that work capacity performance may rely more heavily on aerobic conditioning in these non-competitive participants, as there is substantial difference in overall strength between the two sets of samples. On average, competitive CrossFit athletes reported higher squat (Mn = 163.8 vs. 104.4 kg), press (Mn = 69.1 vs. 46.7 kg), and deadlift (Mn = 187.8 vs. 118.8 kg) maximal strength compared to the participants of the present sample [13,20]. 

The significant changes in physiologic measures of fitness reported in the present study were anticipated and in agreement with previous HIFT research. Several studies have shown significant improvement in aerobic and anaerobic capacity [4], muscular strength [5,6,7,10], and peak power [5]. However, to the authors’ best knowledge, only one other study shows the effects of HIFT on physical work capacity. Drake et al. [7] show that improvements in work capacity may be the largest respective effects of HIFT interventions (ES = 1.06, 95% CI = −0.04, 2.20 Cohen’s *d*). While the present findings report a more modest effect of HIFT on work capacity (ES = 0.412), it may be a function of having a larger and more heterogeneous participant sample than the study performed by Drake et al. [7]. Thus, we contend the effect size reported in this study may be more representative of the true effect of HIFT on physical work capacity. Further, even though the effects on work capacity are only the second largest effects observed in the present study (squat 1RM ES = 0.606), the largest individual variation in effects reported is for work capacity performance (i.e., ranging from 0–107% improvement). Together, these data underscore the potential of work capacity to be considered the primary physiologic outcome of HIFT. 

With work capacity being a central outcome of HIFT interventions, it is important to ask the question of what practical importance this outcome may carry. One of these questions may be to address via what mechanisms HIFT allows for these increases in work capacity. Recently, La Scala Teixeira et al. [22] postulated that functional tasks might challenge the integration and efficiency of body systems in completing a given physical task rather than challenging specific body systems in relative isolation. That is, while running on a treadmill at a high intensity may challenge and develop aerobic capacity, it may do very little to challenge maximal muscle strength. Conversely, if an individual completes a 400 m run then immediately performs 25 box jumps and then repeats this for three rotations as fast as possible (i.e., Table A1, Day 3), it may allow for application of a maximal stimulus to aerobic capacity while also providing a modest challenge to lower extremity muscular strength and/or power. Temporally combining these stimuli may force more efficient system integration to perform the work (i.e., improved economy of effort). The findings of this study provide limited support for this hypothesis, as the association between aerobic capacity and muscular strength are not significant at baseline yet are significantly associated post-intervention. This change in association could point to a shift toward utilizing aerobic metabolism during tasks traditionally thought to be predominantly anaerobic (i.e., maximal strength testing) as a means to allow more complete recovery between work bouts. However, true experimental studies are needed to address this question. Beyond this, determining the practical role of increasing work capacity across various population subgroups should be of particular interest to various exercise practitioners. For example, one might view increasing “work capacity across broad time and modal domains” [8] (p. 37) as a potential means to increase general athletic skill and thus sport performance. However, to date, the authors know of no empirical data to support that increasing physical work capacity in this way improves sport performance. Similarly, one could view increasing physical work capacity as a means to minimize the progression of disability in an individual with a chronic health condition. While studies of HIFT within various clinical populations have been conducted, no investigations to date have looked at the effects of increasing work capacity, specifically, on overall disability [11,12]. Whereas both of these lines of research may prove fruitful, empirical data is needed to identify the potential impact HIFT could have within these populations. Further, determining the effects of different modes of exercise interventions (i.e., aerobic or resistance training vs. HIFT) on work capacity performance may strengthen the position of HIFT as a novel exercise intervention. 

The current work is not without its limitations. First, during the course of data collection our equipment to directly measure oxygen consumption malfunctioned necessitating the use of a prediction equation to determine aerobic capacity. The authors contend that this change contributed to greater observed imprecision in VO_2max_ assessment, ultimately affecting the ability to detect significant change in this measure pre- to post-intervention. Second, work capacity was only assessed within one time domain (i.e., 10 min) and within one specific mode (i.e., WOD). Future research should look to assess work capacity across multiple time domains (e.g., 15 s, 5 min, 10 min, 20 min, and 30 min) and multiple modes (e.g., max deadlifts in 15 s to maximum distance on a rowing ergometer in 30 min). Collecting work capacity data in this way will allow for the development of a “work capacity-time curve” in which the area under the curve (AUC) should be used as the primary outcome measure [8] (p. 35). Taking this more holistic approach may allow for more robust characterization of HIFT outcomes and translation to other lines of research (i.e., sport performance or disability management). Lastly, the present study sample did not allow adequate statistical power within the multiple regression analysis to achieve an acceptable type II error rate (i.e., 0.80). Given the observed ES for the regression analysis, a sample of 77 participants would be needed to achieve the desired type II error rate. However, with the probability of type I error of the overall model being high and the coefficient of determination being low, the authors contend the relationship demonstrated in the present findings will likely hold true for larger samples. 

Future research should emphasize comprehensive assessment (as described above) of work capacity across all studies looking to determine the effect of HIFT on multifactorial participant outcomes (e.g., athletic ability and sport performance). Further, the authors contend that the present study should be replicated to either confirm or refute the conclusions drawn from the present data. These replications should look to design experimental interventions specifically to increase physiologic measures of fitness without intentionally looking to improve work capacity and vice versa. Only through true experimental research designs can any cause-and-effect relationship be investigated and would be welcomed to confirm the independence of physical work capacity from its individual physiologic components. 

## 5. Conclusions 

The present study is the first to demonstrate potential independence of physical work capacity induced by HIFT from changes in associated physiologic measures. These data show significant associations between physiologic measures of fitness and work capacity at baseline assessment along with improvement in these outcomes following a six-week HIFT intervention. However, the observed changes in these measures do not successfully predict the observed change in physical work capacity resulting from the HIFT intervention (i.e., true intervention effects). This independence may point to HIFT operating as a novel exercise modality that improves the integration and efficiency of body systems for producing mechanical work.

## Figures and Tables

**Table 1:** Means, standard deviations, and correlations of primary outcome variables.

	Mn (SD)	WC	VO_2_	Sq	P	DL	PP	FI
Baseline Values (*n* = 25)								
1. Work Capacity (reps)	132.8 ± 32.4	-	-	-	-	-	-	-
2. VO_2max_ (mL/kg^−1^/min^−1^)	43.2 ± 6.9	0.598 **	-	-	-	-	-	-
3. Squat 1RM (kg)	104.4 ± 44.8	0.653 **	0.352	-	-	-	-	-
4. Press 1RM (kg)	46.7 ± 21.3	0.656 **	0.351	0.925 **	-	-	-	-
5. Deadlift 1RM (kg)	118.8 ± 47.8	0.673 **	0.372	0.961 **	0.957 **	-	-	-
6. Peak Power (W)	661.6 ± 258.4	0.571 **	0.407 *	0.893 **	0.939 **	0.890 **	-	-
7. Fatigue Index (%)	57.5 ± 9.6	−0.016	−0.050	0.122	0.336	0.207	0.397 *	-
Post-Intervention Values (*n* = 19)								
1. Work Capacity (reps)	153.5 ± 32.3	-	-	-	-	-	-	-
2. VO_2max_ (mL/kg^−1^/min^−1^)	44.6 ± 7.6	0.799 **	-	-	-	-	-	-
3. Squat 1RM (kg)	109.3 ± 47.5	0.827 **	0.482 *	-	-	-	-	-
4. Press 1RM (kg)	48.0 ± 23.1	0.866 **	0.487 *	0.945 **	-	-	-	-
5. Deadlift 1RM (kg)	124.1 ± 53.1	0.892 **	0.552 *	0.981 **	0.966 **	-	-	-
6. Peak Power (W)	747.8 ± 284.3	0.736 **	0.330	0.905 **	0.846 **	0.872 **	-	-
7. Fatigue Index (%)	59.9 ± 6.6	0.129	−0.056	0.191	0.177	0.193	0.454 *	-

* Significant correlation at *p* < 0.05, ** Significant correlation at *p* < 0.001.

**Table 2:** Multiple regression parameters for predicting change in work capacity (*n* = 19).

Variable	β-Coefficient	Standard Error	95% CI of β	Significance
Overall Model	-	-	-	0.908
ΔVO_2_max (mL/kg^−1^/min^−1^)	0.684	1.28	−1.81, 3.18	0.605
Δ Squat (kg)	−0.395	0.81	−1.97, 1.18	0.638
Δ Press (kg)	−1.068	1.16	−3.33, 1.20	0.379
Δ Deadlift (kg)	0.326	0.52	−0.68, 1.33	0.545
Δ Peak Power (W)	−0.035	0.05	−0.12, 0.12	0.518

## Appendix A

**Table A1:** Detailed description of study high-intensity functional training intervention.

Day	Structure	Workout of the Day *
1	M	Two mile Run (no time cap)
2	GW	(8 Push Press (135/95 lbs) + 8 Pull-Ups) × 5 rounds for time
3	MGW	(12 Goblet Squats (45/25 lbs) + 12 Burpees + 24 Calorie Row) AMRAP in 10 min
4	MG	(400 meter Run + 25 Box Jumps (18/12 inches)) × 3 rounds for time
5	W	Deadlift 5-5-5-5-5 working up to target 85% of 1RM
6	G	Kipping Pull-Up practice for 20 min
7	WM	(10 Thrusters (135/95 lbs) + 100 Double Unders) × 4 rounds for time
8	GWM	(6 Handstand Push-Ups + 12 Deadlifts (185/135 lbs) + 500 meter Row) AMRAP in 12 min
9	GW	(15 Ring Rows + 20 Wall Balls (20/14 lbs)) × 4 rounds for time
10	M	8 km Partner Row (no time cap)
11	W	Front Squat 1-1-1-1-1-1-1-1-1-1 working up to target a 1RM
12	MG	(400 meter Run + 20 Push-Ups) × 5 rounds for time
13	WMG	(5 Cleans (135/95 lbs) + 10 Pull-Ups + 15 Double Unders) AMRAP in 15 min
14	WM	(10/20 − 8/16 − 6/12 − 4/8 − 2/4 repetitions of Power Clean/Calorie Row) for time
15	G	Handstand Push-Up Practice for 20 min
16	W	Squat 3-3-3-3-3-3-3 working up to target 90% 1RM
17	MG	(800 meter Run + 25 Sit-Ups) × 3 rounds for time
18	MGW	(50 Double Unders + 5 Box Jumps (18/12 inches) + 15 Ball Slams (20/14 lbs)) AMRAP in 15 min
19	GW	(6 Strict Pull-Ups + 6 Front Squats (50% Squat 1RM)) × 4 rounds for time
20	M	Two mile Run (no time cap)
21	M	Tabata Double Unders × 2
22	GW	(Maximum repetitions Handstand Push-Ups + 6 Deadlifts (75% 1RM)) × 5 rounds for time
23	GWM	(20 Sit-Ups + 16 Dumbbell Clean and Jerk (45/20 lbs) + 20 Calorie Row) AMRAP in 15 min
24	WM	(30 Kettlebell Swings (45/20 lbs) + 400 meter Run) × 5 rounds for time
25	G	Strict Pull-Up Practice (Loaded) for 25 min
26	G	Muscle Up Practice for 25 min
27	WM	(6 Squats (50% 1RM) + 50 Double Unders) × 4 rounds for time
28	WMG	(12 Goblet Squats (45/25 lbs) + 12 Burpees + 24 Calorie Row) AMRAP in 10 min
29	MG	(400 meter Run + 10 Handstand Push-Ups) × 5 rounds for time
30	W	Clean 1-1-1-1-1-1-1-1-1-1 working up to target 1RM

M = monostructural (i.e., a single exercise modality) exercise, G = gymnastics exercise, W = weightlifting exercise, and AMRAP = “as many rounds as possible.” * WODs were scaled to match individual capabilities on an as needed basis. All scaling options were in accordance with outlined CrossFit scaling practices [8] (p. 75).

## References

[1] Hak P.T., Hodzovic E.H., Hickey B. (2013). The nature and prevalence of injury during CrossFit training. J. Strength Cond. Res..

[2] Serafini P., Hoffstetter W., Mimms H., Smith M., Kliszczewicz B., Feito Y. (2016). Body composition and strength changes following 16-weeks of high-intensity functional training. Med. Sci. Sports Exerc..

[3] Sobrero G.L., Inman C., Stone W., Zagdsuren B., Arnett S.W., Shafer M.A., Lyons S.T., Maples J., Crandall J., Callahan Z. (2015). CrossFit vs. circuit-trained individuals: Effects of a ten-week training program on body composition and bone mineral density. Med. Sci. Sports Exerc..

[4] Zagdsuren B., Sobrero G., Inman C., Arnett S., Schafer M., Lyons S., Maples J., Crandall J., Callahan Z. (2015). CrossFit vs. circuit-training: Effects of a ten-week training program on aerobic, anaerobic, and flexibility indicators. Med. Sci. Sports Exerc..

[5] McKenzie M.J. (2015). CrossFit improves measures of muscular strength and power in active young females. Med. Sci. Sports Exerc..

[6] Brown J.T., Sobrero G.L., Inman C., Stone W., Zagdsuren B., Arnett S.W., Schafer M.A., Maples J., Crandall J., Callahan Z. (2015). CrossFit vs. circuit-trained individuals: Effects of a ten-week training program on muscular strength and endurance. Med. Sci. Sports Exerc..

[7] Drake N.B., Smeed J., Carper M.J., Crawford D.A. (2017). Effects of short-term CrossFit training: A magnitude-based approach. J. Exerc. Physiol. Online.

[8] (2017). CrossFit Level 1 Training Guide.

[9] Haddock C.K., Poston W.S.C., Heinrich K.M., Jahnke S.A., Jitnarin N. (2016). The benefits of high-intensity functional training fitness programs for military personnel. Mil. Med..

[10] Heinrich K.M., Spencer V., Fehl N., Poston W.S.C. (2012). Mission Essential Fitness: Comparison of Functional Circuit Training to Traditional Army Physical Training for Active-Duty Military. Mil. Med..

[11] Heinrich K.M., Becker C., Carlisle T., Gilmore K., Hauser J., Frye J., Harms C.A. (2015). High-intensity functional training improves functional movement and body composition among cancer survivors: A pilot study. Eur. J. Cancer Care.

[12] Heinrich K.M., Patel P.M., O’Neal J.L., Heinrich B.S. (2014). High-intensity compared to moderate-intensity training for exercise initiation, enjoyment, adherence, and intentions: An intervention study. BMC Public Health.

[13] Butcher S., Neydely T., Horvey K., Benko C. (2015). Do physiological measures predict selected CrossFit benchmark performance?. Open Access J. Sports Med..

[14] Dankel S.J., Buckner S.L., Jessee M.B., Mouser J.G., Mattocks K.T., Abe T., Loenneke J.P. (2018). Correlations do not show cause and effect: Not even for changes in muscle size and strength. Sports Med..

[15] Bruce R., Hosmer D. (1973). Maximal oxygen intake and nomographic assessment of function aerobic impairment in cardiovascular disease. Am. Heart J..

[16] Foster C., Jackson A.S., Pollack M.L., Taylor M.M., Hare J., Sennett S.M., Rod J.L., Sarwar M., Schmidt D.H. (1984). Generalized equations for predicting functional capacity from treadmill performance. Am. Heart J..

[17] Bar-Or O. (1987). The Wingate Anaerobic Test an update on methodology, reliability and validity. Sports Med..

[18] McGuigan M., Haff G.G., Triplett N.T. (2016). Principles of test selection and administration. Essentials of Strength Training and Conditioning.

[19] Beaver W.L., Lamarra N., Wasserman K. (1981). Breath-by-breath measurement of true alveolar gas exchange. J. Appl. Physiol..

[20] Serafini P.R., Feito Y., Mangine G.T. (2017). Self-reported measures of strength and sport-specific skills distinguish ranking in an international online fitness competition. J. Strength Cond. Res..

[21] Bellar D., Hatchett A., Judge L.W., Breaux M.E., Marcus L. (2015). The relationship of aerobic capacity, anaerobic peak power and experience to performance in HIT exercise. Biol. Sport.

[22] La Scala Teixeira C.V., Evangelista A.L., Novaes J.S., Da Silva Grigoletto M.E., Behm D.G. (2017). “You’re only as strong as your weakest link”: A current opinion about the concepts and characteristics of functional training. Front. Physiol..

